# Assessment of pulmonary function among cleaners in governmental hospitals in Addis Ababa, Ethiopia; comparative cross-sectional study

**DOI:** 10.1186/s13104-019-4401-2

**Published:** 2019-07-08

**Authors:** Biruk Getahun, Diresibachew W. Haile

**Affiliations:** 1 Department of Medical Physiology, School of Medicine, College of Medicine and Health Sciences, Bahir Dar University, P.O.Box 79, Bahir Dar, Ethiopia; 2 Department of Medical Physiology, School of Medicine, College of Health Sciences, Addis Ababa University, Addis Ababa, Ethiopia

**Keywords:** Body mass index, Spirometry, Pulmonary function tests, Cleaners, Hospital

## Abstract

**Objective:**

Work-related disorders are the major causes of different diseases in working populations. Respiratory disorders are among the most common occupational diseases. The alterations of the pulmonary function of professional cleaners in hospitals have not been addressed previously in Ethiopia. The aim of this study was to assess the pulmonary functions of cleaners working in the hospital setting in Addis Ababa, Ethiopia. A comparative cross-sectional study design was employed. The study was composed of 70 cleaners and 70 control subjects. Spirometer was used to measure pulmonary function. The data were coded, entered and analyzed using SPSS version 20.

**Results:**

The mean and standard deviation of both actual value and percent predicted for forced vital capacity, forced expiratory volume in 1 s, percent ratio of forced vital capacity and forced expiratory volume in 1 s, peak expiratory flow rate and forced expiratory flow between 25 and 75% of cleaners were found to be significantly lower than the control group. Among cleaners 24.3% (n = 17) and controls 8.6% (n = 6) had shown obstructive lung disease, and among cleaners 22.9% (n = 16) and controls 4.3% (n = 3) had shown restrictive pattern.

## Introduction

Work-related exposures cause diseases in worker populations [1]. An International Labor Organization (ILO) report estimated that 2 million occupational fatalities occur across the world every year [2]. Occupational lung diseases are one of the major causes of respiratory morbidity and mortality especially in developing countries where the majority of workers work without proper protection [3]. Occupational exposures to dust, fumes, chemicals, and gases are associated with an increased prevalence of respiratory symptoms and impairment of lung function [4]. Exposure to dust has long been associated with the prevalence of varying degrees of airway obstruction and respiratory symptoms in human [5].

Inhalation of dust and cleaning agents can cause the lungs to react in a wide variety of ways, irritating the airways, exacerbating the conditions such as asthma and setting up an inflammatory reaction and fibrosis [6]. Work-related asthma (WRA) and occupational chronic obstructive pulmonary disease (COPD) are serious and sometimes fatal diseases, which can lead to ill health, inability to work and lost productivity [7].

Cleaning is one of the high-risk occupations which may cause different diseases among workers. Respiratory disorders are among the most common occupational diseases of cleaners. Exposure to different types of airways irritants (gases and aerosols) from cleaning agents over time might lead to COPD development among cleaners [8, 9].

Although cleaners represent a significant part of the working population worldwide, they remain a relatively understudied occupational group [10]. There is a scarcity of literature regarding the prevalence of COPD in occupational cleaners [8] though occupational exposures and COPD are described for other vocations [11, 12]. At present, the standards and norms for the management of wastes in industrialized countries have substantially reduced the occupational health impacts [13]. In developing countries, however, the health-related underpinnings of solid waste management still need to be addressed. For instance, workers manually collect wastes [13].

Even though great attention has been given to different occupations on health, pulmonary function complications of cleaners have been poorly characterized. The alterations of the pulmonary function of professional hospital cleaners have not been addressed previously in Ethiopia. Therefore, the study has attempted to evaluate lung functions among hospital cleaning workers comparing with the control group. The result of the study has the potential to reduce morbidity/mortality, by increasing awareness of cleaners on cleaning-induced pulmonary complications.

## Main text

### Materials and methods

The comparative cross-sectional study design was employed to assess pulmonary function among randomly selected female cleaners and non-cleaners who works at three governmental hospitals (Tikur Anbessa, Yekatit 12 and Zewditu) in Addis Ababa city. Non-cleaners were administrative workers.

The sample size calculation was performed for percent predicted FEV_1_, with medium effect size of E = 6.5, a power of β = 0.80, and an α = level of 0.05 [14, 15]. A sample size of 70 for each group was proposed, including a 10% increment to account for non-response. The study participants were selected by systematic random sampling. Registration book of workers was used as a frame to select the participants.

Both groups were non-smokers and aged between 18 and 64 years. For both study and control groups, those with tuberculosis, obesity (BMI ≥ 30), pregnancy, heart failure, ascites, abdominal tumor, recent surgery of thorax, abdomen, oral, self-reported or known diagnosis of lung or chest wall disease like asthma, chronic bronchitis, pneumothorax, common cold, chronic cough; history of smoking, any acute illness and abdominal pain of any cause were excluded from the study. The socio-demographic data was collected using an interview. A standardized questionnaire based on British Medical Research Council Questionnaire (BMRC) was prepared and used during face-to-face interview. Pulmonary function tests and anthropometric measurements were performed on 70 cleaners and 70 administrative workers (controls). Height with the subject standing barefoot, and weight of each subject were measured using the scale to the nearest 1 cm and 1 kg, respectively. Age was recorded to the nearest year.

Respiratory function parameters (FVC, FEV_1_, FEV%, PEFR, and FEF_25–75%_) were measured using spirometer (“spiropro^®^”—JAEGER) in accordance with recommendations of the American thoracic society (ATS). The apparatus was calibrated for each procedure. Three maneuvers were performed per a subject and then one of the maximum value of the three trials was used for analyses.

### Statistical analysis

Data were coded, entered, cleaned and analyzed using Statistical Package for Social Science (SPSS) version 20. Descriptive statistics and independent samples t-test were conducted in the data analysis and interpretation.

Spirometry values were categorized in obstructive, restrictive and mixed patterns according to ATS/ERS guidelines. %predicted value was calculated dividing observed value by predicted value and multiplying by 100 and this value was automatically calculated by the spirometer.

### Results

#### Socio-demographic characteristics

The study comprised of 140 participants and all were females, aged 20–45 years. Both case and control groups were proportional in age, height, weight, and body mass index (Table 1).

**Table 1:** Socio-demographic and anthropometric characteristics of the study participants at governmental hospitals in Addis Ababa, Ethiopia

Variables	Exposed group (n = 70) frequency	Control group (n = 70) frequency	
Level of education			
Non-formal	1 (1.4%)	0	
Primary school	30 (42.9%)	3 (4.29%)	
Secondary school	34 (48.6%)	18 (25.71%)	
Diploma	5 (7.1%)	34 (48.6%)	
1st degree and master	0	15 (21.43%)	
Service in years			
One to three	39 (55.7%)	36 (51.4%)	
Four to six	18 (25.7%)	20 (28.6%)	
Seven to nine	8 (11.4%)	7 (10%)	
Above nine	5 (7.1%)	7 (10%)	

	Mean ± SD	Mean ± SD	P-value
Age (years)	28.00 ± 7.40	28.33 ± 7.44	0.79
Height (cm)	154.69 ± 6.45	156.64 ± 5.97	0.06
Weight (kg)	54.34 ± 7. 71	55.70 ± 8.46	0.32
BMI (kg/m^2^)	22. 68 ± 3. 26	22.66 ± 2.96	0.97

n, number; SD, standard deviation; BMI, body mass index; cm, centimeter; kg, kilogram; m, meter

#### Pulmonary function tests

An independent sample t-test was conducted to compare lung function measurements of exposed and control subjects. Accordingly, the mean actual and percent predicted values of forced vital capacity (FVC), forced expiratory volume in 1 s (FEV_1_), percent ratio of forced vital capacity and forced expiratory volume in 1 s (FEV_1_/FVC%,), peak expiratory flow rate (PEFR) and forced expiratory flow between 25 and 75% (FEF_25–75%_) were lower in cleaners than the control group (Table 2). As it is revealed in the table below, there were statistically significant differences on the pulmonary function parameters (FVC, FEV_1_, FEV_1_/FVC%, PEFR, and FEF_25–75%_) for both actual and % predicted values between the cleaners and control group (P < 0.05) (Table 2).

**Table 2:** An independent sample t-test result for comparison of pulmonary function parameters between cleaners and control group at governmental hospitals in Addis Ababa, Ethiopia

Variables	Exposed group (n = 70) mean ± SD	Control group (n = 70) mean ± SD	95% CI	P-value
FVC (L)				
Actual V.	3.11 ± 0.75	3.58 ± 0.72	0.22–0.71	0.000**
%Predicted V.	92.8 ± 21.9	101.9 ± 16.4	2.65–15.6	0.006*
FEV_1_ (L/s)				
Actual V.	2.52 ± 0.69	3.05 ± 0.57	0.31–0.74	0.000**
%Predicted V.	87.1 ± 21.8	99.7 ± 18.7	5.75–19.3	0.000**
FEV_1_/FVC (L/s)				
Actual V.	81.4 ± 13.16	85.9 ± 11.3	0.43–8.62	0.030*
%Predicted V.	96.5 ± 15.9	103.7 ± 13.6	2.3–12.2	0.004*
PEFR (L/s)				
Actual V.	3.82 ± 1.43	4.72 ± 1.55	0.41–1.41	0.000**
%Predicted V.	60.5 ± 21.5	72 ± 22.5	4.20–8.90	0.002*
FEF_25–75%_ (L/s)				
Actual V.	2.66 ± 1.18	3.64 ± 1.14	0.59–1.36	0.000**
%Predicted V.	80.7 ± 34.5	105.3 ± 36.5	12.8–36.5	0.000**

SD, standard deviation; CI, confidence interval; V., value

* P-value < 0.05, ** P-value < 0.001

**Fig. 1:**
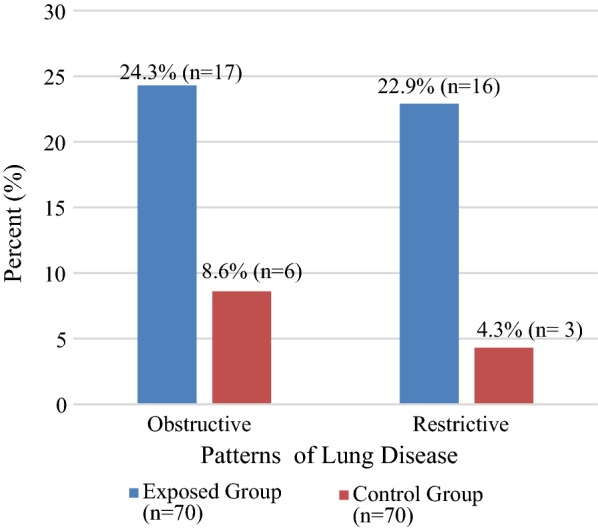
Results of the descriptive statistics of patterns of pulmonary function impairment among cleaners and control groups at governmental hospitals in Addis Ababa, Ethiopia

Patterns of pulmonary function impairment between the exposed and control groups were compared using descriptive statistics. About 17 (24.3%) of cleaners developed the obstructive type of lung disease whereas 16 (22.9%) of them developed restrictive lung disorder. The disorder among cleaners was very high as compared to the controls where 6 (8.6%) and 3 (4.3%) of them developed obstructive and restrictive lung disorders, respectively (Fig. 1).

### Discussion

The results of this study showed a comparable mean of age, height, weight, and BMI of both cleaners and control groups. There was no significant difference between these variables between the two comparable groups so that both groups were relatively comparable.

An independent sample t-test of this study showed that the mean actual and percent predicted values of pulmonary function parameters (FVC, FEV_1_, FEV_1_/FVC%, PEFR, and FEF_25–75%_) of cleaners were significantly lower than non-exposed groups.

Hence, in the present study, both the obstructive and restrictive patterns of pulmonary function impairments of the study groups (cleaners and controls) were identified, but the mixed pattern was not seen in both groups. Accordingly, about 24.3% (n = 17) among cleaners and 8.6% (n = 6) among controls had actual FEV_1_/FVC% less than 70% and FEV_1_ percent predicted less than 80% which is an indication of obstructive pattern of pulmonary function impairment. About 22.9% (n = 16) among cleaners and 4.3% (n = 3) among controls had actual FEV_1_/FVC% greater than 70% and FVC percent predicted less than 80% indicating restrictive pattern of pulmonary function impairments. As a result, the prevalence of Obstructive and restrictive patterns of pulmonary function impairments was higher among cleaners. Therefore, cleaners have a higher opportunity to develop these patterns of pulmonary function impairment (obstructive and restrictive) as long as they became exposed to cleaning agents and dust in cleaning. These may be due to the fact that high exposure to different sensitizing and irritating cleaning chemicals and dust from the surface of the floor increase the opportunity to develop these patterns of pulmonary function impairment among cleaners than the control groups.

These results were in line with the results of several studies done by different scholars. A study conducted in Iran among garbage collectors presented a significantly decreased mean percent predicted value of FEV_1_ and FEV_1_/FVC% than controls [16]. Another study conducted on impairment of lung functions in adult sweepers reported significantly reduced actual and predicted values of FVC, FEV_1_, FEV_1_/FVC%, PEFR and FEF_25–75%_ among cleaners than controls [6].

The study conducted among sanitary workers showed significantly decreased mean of the actual values of FVC, FEV_1_, FEV_1_/FVC%, PEFR, and FEF_25–75%_ when compared to control groups. The mean predicted values of FVC, FEV_1_, PEFR, and FEF_25–75%_ were also significantly reduced when compared to control groups except for FEV_1_/FVC% [3]. But FEV_1_/FVC% was significantly reduced in the present study. A study done on female sweepers in India [17] found significantly decreased values of FVC, FEV_1_, and PEFR among sweepers as compared with their matched controls.

A study conducted in India [18] on acute lung function response to dust in street sweepers revealed that the mean values of FVC, FEV_1_, PEFR, and EFE_25–75%_ were significantly decreased in sweepers as compared with their matched controls. Unlike our finding, however, this study has not found a statistically significant reduction in FEV_1_/FVC%. Hence, our study substantiates the assumption that exposure to cleaning agents in hospital cleaning workers has a detrimental effect on the lung function.

### Conclusion

This study has assessed the pulmonary functions of cleaners working at a hospital setting. It has attempted to evaluate lung functions among hospital cleaning workers comparing with the control group.

As the results of the study has revealed there was a significant reduction in FVC, FEV_1_, FV_1_/FVC%, PEFR and FEF_25–75%_ among cleaners. Both obstructive and restrictive lung disease patterns were highly prevalent among cleaners. The study has the potential to reduce morbidity/mortality, by increasing awareness of cleaners on cleaning-induced pulmonary function impairments.

## Limitations

Finally, our study is not without limitations. Selection of sample for exposed and control groups were based on the health history information. The study did not include all age groups rather it was limited to age range of 20–45 years which was because of the age variability of cleaners. However, this age range would have been more representative of the general age proportion of the study population. The study did not include both sexes, and this is not purposive rather it is because of the availability of cleaners. Therefore our findings are still relevant and valid in drawing attention to the population.

## Data Availability

All data analyzed during this study are available from the corresponding author on reasonable request.

## Abbreviations

ATS: American Thoracic Society
BMI: body mass index
BMRC: British Medical Research Council Questionnaire
CI: confidence interval
COPD: chronic obstructive pulmonary disease
ERS: European Respiratory Society
FEF: forced expiratory flow
FEV_1_: forced expiratory volume at one second
FVC: forced vital capacity
PEFR: peak expiratory flow rate
PFT: pulmonary function test
SD: standard deviation
SPSS: Statistical Package for Social Science
WRA: work-related asthma
PFT: pulmonary function test
